# Air Quality Effects on Human Health and Approaches for Its Assessment through Microfluidic Chips

**DOI:** 10.3390/genes8100244

**Published:** 2017-09-27

**Authors:** Frank Schulze, Xinghua Gao, Darius Virzonis, Samar Damiati, Marlon R. Schneider, Rimantas Kodzius

**Affiliations:** 1 German Federal Institute for Risk Assessment (BfR), German Centre for the Protection of Laboratory Animals (Bf3R), 10589 Berlin, Germany; marlon.schneider@bfr.bund.de; 2 iSmart, Materials Genome Institute, Shanghai University (SHU), Shanghai 201800, China; gaoxinghua@t.shu.edu.cn; 3 Department of Electrical Engineering, Kaunas University of Technology, 35212 Panevezys, Lithuania; darius.virzonis@ktu.lt; 4 Department of Biochemistry, King Abdulaziz University, Jeddah 80203, Saudi Arabia; sdamiati@kau.edu.sa; 5 Institute for Synthetic Bioarchitecture, Department of Nanobiotechnology, University of Natural Resources and Life Sciences, 1190 Vienna, Austria; 6 Mathematics and Natural Sciences Department, The American University of Iraq, Sulaimani, Sulaymaniyah 46001, Iraq; rimantas.kodzius@auis.edu.krd

**Keywords:** air pollution, particulate matter (PM), PM2.5, microchip, organ-on-chip (OoC)

## Abstract

Air quality depends on the various gases and particles present in it. Both natural phenomena and human activities affect the cleanliness of air. In the last decade, many countries experienced an unprecedented industrial growth, resulting in changing air quality values, and correspondingly, affecting our life quality. Air quality can be accessed by employing microchips that qualitatively and quantitatively determine the present gases and dust particles. The so-called particular matter 2.5 (PM2.5) values are of high importance, as such small particles can penetrate the human lung barrier and enter the blood system. There are cancer cases related to many air pollutants, and especially to PM2.5, contributing to exploding costs within the healthcare system. We focus on various current and potential future air pollutants, and propose solutions on how to protect our health against such dangerous substances. Recent developments in the Organ-on-Chip (OoC) technology can be used to study air pollution as well. OoC allows determination of pollutant toxicity and speeds up the development of novel pharmaceutical drugs.

## 1. Introduction

The planet Earth is the only known biosphere for human beings; it is, therefore, of paramount importance for human health to protect and preserve this environment. For this, it is imperative to detect pollution sources, understand contaminants and the effects these substances may have on our body after both short and long-term exposure (or in other words, to understand acute and chronic toxicity). There are various particles, chemicals, and gases that influence the quality of the air we breathe. Incomplete combustion of coal, diesel, or other fossil fuels, can lead to the formation of cancerogenic substances. While providing numerous useful and versatile compounds in high amounts, such as plastics or novel chemicals, the corresponding industry often introduces these materials without prior testing for the long-term effects on the environment and human health. One notorious example for the unintentional introduction of hazardous particles by the industry is the fabrication and further incorporation of asbestos into building materials [1]. Similar scenarios occurred with many chemicals that are classified as volatile organic compounds (VOC). More recently, the advent of nanomaterials helped to create many novel materials and to improve the properties of already existing ones. These materials include, but are not restricted to, carbon-derivates, organic and inorganic nanoparticles, 2D-materials such as graphene, and others. Here, not only the chemical composition, but also the size and shape of the nanomaterial, all have an impact on human health. These novel materials, before being released for public use, and thus, into the environment, need to be tested for their toxicity. This includes time and cost-effective physiochemical analysis of different pollutants, and subsequent investigations on possible health risks. In this context, recent advances in fabrication techniques might contribute, as they allow for the miniaturization of sensors and detectors, and the design of organ-like in vitro systems.

**Figure 1:**
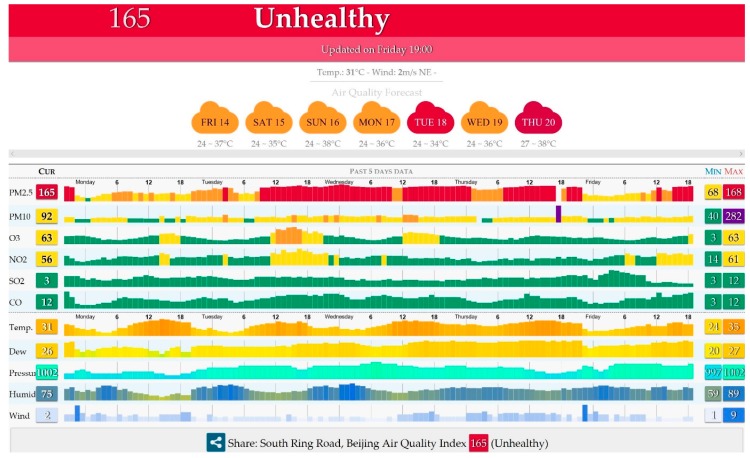
An example of the information provided by air monitoring stations in Beijing, China. Besides the usual temperature, dew, pressure, humidity, and wind values in hourly readings, the information on following pollutants can be seen: particular matter 2.5 (PM2.5), PM10, O_3_, NO_2_, SO_2_, and CO. Here, the unhealthy status is based on current highest PM2.5 values. The figure is reproduced with permission [11].

The advance of microelectromechanical systems (MEMS), a basis for the fabrication of microchips, was initiated by the need to concentrate many functional structures within a small area [2]. The micrometer- and even nanometer-sized structures are made by different techniques, such as photolithography, injection molding, embossing, etching techniques, or 3D printing [3]. For the fabrication of MEMS, different materials such as silicon, metals, ceramics, or polymers can be used. The applications for the MEMS devices range from ultrasound transducers [4,5] and Lab-on-Chip (LoC) [6] to rather sophisticated Organ-on-Chip systems (OoC) [7]. Usually, the chip itself represents the core of a given system, while the accompanying peripheral instrumentation setup can be much larger. For example, in air quality sensors, samples of air are first taken and then delivered to the chip component of the setup. The particle size and chemistry is then determined on the chip, providing an immediate readout. Similarly, cells ranging in numbers between one and a few hundred to thousands can be deposited and examined using MEMS technology. Here, the chip is part of a setup that usually involves a microscope, fluid control, and sensors, with their corresponding electronics, needed to deliver the readout of interest. For most portable devices, the chips are optimized towards an increase in energy efficiency. There are countless possibilities to utilize LoC and OoC systems. The established technologies already allow for fabrication and subsequent application, and are robust enough to be used in extraterrestrial space, for example in satellites [8]. For the use in space, MEMS allow minimizing various components down to a size range of a few micro- or nanometer-microsensors, actuators, accelerometers, heat controllers, microfluidic thrusters, microwave devices, satellite communication, and others. This facilitates the development of highly reliable, economical and mass-produced satellites that might also be used for monitoring pollutant sources, such as dust storms or volcanic activity [9]. On the ground, LoC systems offer an unprecedented sensitivity in the assessment of small particle characteristics or the detection of chemicals. For example, hazardous particles in the air, which can originate from pollution sources, can be assessed by the right type of device [10]. Nowadays, it is not uncommon to access the readouts of the closest air monitoring station through a respective online portal. The readouts are determined and immediately provided in real-time (Figure 1) [11]. The air monitoring stations cost from several hundred to tens of thousands of dollars, and are usually affordable for cities with a population of over one hundred thousand people [12].

Conventional 2D cell culture, although of immense value in past research, is strongly limited in recreating tissue-specific parameters, such as pH, pO_2_, extracellular matrix composition, or the presence of mechanical forces. Thus, the translation of data gained in conventional cell culture into complex organisms is hampered, a disadvantage that is apparent in many fields, such as regenerative medicine or toxicology. To compensate for these shortcomings, animal experiments are widely used, especially in the risk assessment or testing of chemicals and novel drugs. Yet, the species-specific differences between test animals and humans make a direct translation of results difficult [13,14]. One possible way to overcome these shortcomings is the use of OoC systems. Here, microfluidic platforms or chips are used to recreate a physiological environment that resembles the specifics of the tissue of interest, and also allows the co-culture of different cell types in 2D or 3D [15]. In addition, the connection of several different organ-models on one platform was proposed, a concept dubbed multi-organ or body-on-a-chip. OoC systems, like functional Lung-on-Chip, Heart-on-Chip, Kidney-on-Chip, Liver-on-Chip and other OoCs are readily available, and funding in this area of research is increasing (Table 1) [16]. These developments raise big hopes, as they allow for low-cost testing of drugs and chemicals, while physiochemical and cellular parameters of the tissue of interested can be recreated, therefore increasing the predictive value of the OoC. Recent lung-on-a-chip systems allow for testing airborne particles and substances for their toxicity on various cells in a monolayer or as 3D cell aggregates [17]. One example of such an approach is the smoking small airway-on-a-chip where a device that mimics human smoking is connected to a lung-on-a-chip to induce and investigate pathological phenotypes caused by inhaling cigarette smoke [18].

In this review, we want to provide an overview on air pollutants, including their sources and accompanied health risks due to exposure. Furthermore, the usage of LoC and OoC devices in the context of air quality assessment and health risk investigation will be discussed.

## 2. The Air We Breathe

Over the Earth’s history, the atmosphere’s gas composition has changed considerably. Already some 3.5 billion years ago, photosynthetic archaea and bacteria started to produce oxygen (O_2_), resulting in a significant accumulation in the Earth’s atmosphere within the last two billion years [19]. In the course of this vast time frame, the oxygen level rose up to 35% by volume [20], compared with 21% today. Since the last peak of oxygen (the last 300 million years to the present), the Earth’s atmosphere has retained an almost constant composition [21].

The composition of air that is considered as clean consists mainly of three gases and some water vapors. Besides the three main air components, nitrogen (N_2_, 78.084%), oxygen (O_2_, 20.964%), and carbon dioxide (CO_2_, 0.0407%), there is a small percentage of other trace gases in the air, such as argon (Ar), neon (Ne), helium (He), hydrogen (H_2_), krypton (Kr), nitrous oxide (NO_2_), ozone (O_3_), and methane (CH_4_) [22,23]. The amount of water vapor may occupy 1% of the air at sea level, or even up to 5% by volume in humid and hot environments. Additionally, the air contains some other aerosols, such as colloids of fine solid particles or liquid droplets. These aerosols may originate from non-anthropogenic sources (such as sea salt, pollen, spores, dust), or from anthropogenic activities (such as cigarette smoke, fumes in car exhaust, industrial air pollution, etc.).

## 3. Air Pollution Sources

Air is not only composed of gases or aerosols. In fact, air contains everything light enough to be lifted from the Earth’s surface, including lightweight pollutants that are emitted by human activity. While the human organism is adapted to breathe air to allow oxygen uptake into the bloodstream, the exposure to gases or particles from anthropogenic origin may cause discomfort, allergic reactions, or even more serious problems such as the formation of cancer as a result of long-term exposure. Many factors contribute to the damaging effects of pollutants to human health: the chemical composition, the size, shape, exposure time and intensity, and negative synergisms when combined with other harmful substances. Not only humans, but also animals and plants (such as food crops) can suffer from contaminated air. There are also some positive effects that can arise from air pollutants—dust particles in the air may deliver nutrients like phosphorus (P), nitrogen (N), and iron (Fe), to ecosystems with nutrient-poor soils (e.g., The Arabian Gulf) [24,25,26].

### 3.1. Indoor Air Pollution Origins and Prevention

An Environmental Protection Agency (EPA) study showed that people in the USA spend most of their time indoors (up to 93% of the total time, with 6% in automobiles) [27]. In analogy to atmospheric pollution, it is therefore important to understand and control indoor air pollution [28,29,30]. The indoor air may be contaminated from pollution sources outside the building, but might also originate from materials inside, such as building materials, furnishings, rugs and carpets, and some aerosols from personal care products. Additionally, in developing countries, pollutants in buildings can originate from burning coal, or wood for cooking and heating [31,32]. It is relatively easy to detect indoor pollutants and control their levels by filtration and ventilation. Other potential airborne hazards for humans within buildings include an increased concentration of some gases (such as carbon monoxide (CO), CO_2_, O_3_, or Radon (Rn)), the presence of organic compounds, asbestos, and even high concentrations of microorganisms.

#### 3.1.1. Gaseous Pollutants

Carbon monoxide (CO) and dioxide (CO_2_) can originate from tobacco smoke or the combustion of fossil fuels. CO is formed in the case of incomplete combustion. Raised levels of CO and CO_2_ can deplete oxygen levels in the air, leading to nausea, drowsiness, headaches, unconsciousness, and eventually death. Another source for increased CO_2_ levels is human metabolic activity, especially with many occupants enclosed in one room [33]. The control of CO and CO_2_ concentrations can be facilitated by implementing a smoke-free environment and plenty of outdoor air ventilation—either by opening the windows or adjusting ventilation levels [34,35]. Ventilation systems with integrated CO_2_ sensors can adjust the ventilation intensity according to the actual CO_2_ levels.

Atmospheric O_3_ is formed from free oxygen radicals [36]. O_3_ is produced by the sun’s ultraviolet light, lightning, air purifiers with ionizing function, and some devices designed to kill bacteria and sterilize [37]. Also, high voltage devices, such as laser printers, tasers, arc welders, and electric motor sparking can generate significant levels of ozone. Usually, the highest concentrations of ozone can be found in the so-called ozone layer that is situated on the top of the stratosphere, around 20 to 30 km above the Earth. Ozone is a very powerful oxidant, damaging the mucous and respiratory tissues in humans and animals. Additionally, some chemicals used for cosmetics (such as skin oil) or cleaning (based on terpene or citrus extracts) may react with ozone, and eventually produce toxic byproducts or even dangerous nanoparticles. Ventilation might fail in reducing indoor ozone levels, as the atmospheric air may be a major source of ozone [38]. A potential solution would be to install ozone filters in ventilation systems. Some larger airplanes have ozone filters to prevent the introduction of ozone from the outside [39].

Radon (Rn) is a common gaseous pollutant, and a pervasive and serious hazard in an indoor environment. The two most common radioactive elements on Earth are thorium (Th) and uranium (U). Radon is the by-product of the radioactive decay of Th and U to the intermediate product, radium (Ra). Radon is present in various rocks, such as uranium ores, shales, granite, and even limestone, and accumulates in high quantities in mines [40,41]. Some hot springs and spring waters may contain a high concentration of radon too [42]. Usually, radon enters a building through fundaments, or originates from the building material itself (such as granite). Radon tends to accumulate at the ground level, as it is a heavy gas. Radon mitigation (reducing radon levels) is done by sealing the basement, thus filling any cracks present, and of course, by enough ventilation, possibly with positive pressurization [43,44]. The half-life of radon is only 3.8 days. Therefore, removing or isolating the source will greatly reduce the hazard within a few weeks. There are kits and even digital radon detectors and various sensors available. Radium and its decay product, ^222^Rn, are classified as carcinogens to humans [45]. When gaseous, radon is even more dangerous, as it can be inhaled directly into the lungs. Lung cancer is the main illness caused by radon exposure, even for non-smokers [46,47,48,49,50]. Some studies point to a synergetic effect of smoking and radon exposure [50]. Furthermore, there are some hints, also inconsistent, in the published literature on the correlation between radon exposure and chronic lymphocytic leukemia [51].

#### 3.1.2. Organic Compounds

The so-called volatile organic compounds (VOC)s are organic chemicals, with a low boiling point causing them to evaporate at rather low temperatures. There are many different VOCs, with some perceived as scents or odors, such as formaldehyde, chloroform, benzene, acrolein, acetaldehyde, and other gases emitted from certain solids and liquids. Similar to the case of radon gas, the indoor concentration of VOCs usually exceeds the outside ones several times. There are many household products emitting VOCs. Furniture wood, many paints, and adhesives release formaldehyde (H_2_CO). An increase in temperature and a relatively high humidity can speed up the vaporization of formaldehyde from wood-based materials [52]. Overheated cooking oil emits not only formaldehyde, but also acrolein. Formaldehyde affects the mucous membrane, causing discomfort [52].

Benzene (C_6_H_6_) is a cancerogenic VOC, emitted mostly by tobacco smoke, from car exhausts or stored fuels. It is a additive for gasoline (petrol), supplemented by up to 1%, thereby increasing the octane rating and reducing knocking.

Benzene is heavier than air, so after evaporation, it descends towards low-lying areas near the ground. In humans, benzene is rapidly metabolized in the liver and excreted via the urinal tract [52]. Possible routes of exposure may be found in breathing contaminated air or by drinking soft drinks supplemented with plenty of benzoic acids and ascorbic acid [53].

Chloroform (CHCl_3_) is classified as an extremely hazardous substance and can be released from hot chlorinated water at home. The exposure routes include inhalation, and oral or dermal absorption. Chloroform affects mainly the central nervous system (CNS) [54]. 

There are many other VOCs, released even from office equipment, such as laser copiers and printers, carbonless copy paper, permanent markers, and glues or adhesives [55]. Some microbes, like bacteria and fungi (e.g., *Stachybotrys chartarum*) release volatile organic compounds (called microbial VOCs (MVOCs), or mushroom alcohol) [56], which are linked to sick building syndrome [57]. The symptoms of a smelly moldy house are similar to those of allergenic responses—running nose, watery eyes, itchiness, and rash. Introducing regulations to furniture, paints and other products manufacturers help to limit the exposure to VOCs indoors (for example by substituting organic to aqua-based paints). Also, the availability of products that emit only low concentrations of VOC is constantly increasing.

The consumer should buy products that contain only low levels of VOCs, or none at all. Of course, proper ventilation helps to remove VOCs from indoor areas in buildings. There are even VOC sensors in ventilation systems, helping to increase ventilation speed, depending on the VOC concentration in the air. Some conditioning systems are designed to absorb and eliminate VOCs from the room. Interestingly, some cancer types can be detected by measuring the emitted VOCs (hundreds of different compounds) from the lungs [58].

#### 3.1.3. Asbestos

One of the most dangerous building materials is asbestos, which was extensively used in the past, especially in buildings built before 1975. Asbestos was one of the most common isolation and insulation materials available. Asbestos is made of silicate material [1,59,60], and is usually stable when kept in place and not being disintegrated. Drilling or removing isolation containing asbestos results in damaging and subsequent breakdown of the material, thereby releasing microscopic fibers. These asbestos fibers are much smaller than fine sand, and some even smaller than bacteria (in the size of 0.1–100 µm). The particular asbestos needle-like shape with sharp angles contributes to the lodging of asbestos into the lungs [61]. The long-term exposure and inhalation of asbestos contribute to the development of lung cancer, in particular, to its specific form, termed mesothelioma [62,63]. Smokers have an even higher risk of lung cancer if additionally exposed to asbestos. Currently, asbestos has been banned in most countries. Some substitute materials are suggested and used, such as fiberglass, polybenzimidazole (PBI) filter, and mineral or glass wool. Asbestos can be destroyed by thermal decomposition at 1000 °C or over 1250 °C, resulting in the formation of silicate glass [64].

#### 3.1.4. Biological Pollution Sources

Indoor pollutants originating from biological sources play a significant role in our environmental health. 

Microorganisms can be pathogenic or non-pathogenic, depending on their ability to challenge or evade the host immune system. Further, the relationship between host and microorganism shifts from mutual to unilateral, in the case of pathogenicity. A vast number of non-pathogenic microorganisms are regularly residing on and inside our body (also called microbiota), where both the microorganisms and our body benefit from such a relationship. However, immune-comprised individuals, or persons with an immunodeficiency, are especially susceptible to infections, as pathogenic bacteria gain access to the body’s interior. Furthermore, microorganisms that are considered non-pathogenic might turn pathogenic when the host immune system is not able to prevent their overgrowth. From the ≈1400 known pathogenic species in total, around one hundred are pathogenic bacteria capable of causing infectious diseases in healthy humans [65]. Since microorganisms can be propagated through air, they are classified and treated as air pollutants that exhibit a hazard and have exposure thresholds.

Not only plant pollen or animal dander can be the cause of asthma and allergies, but also microorganisms, like certain bacteria and molds. Improper ventilation and moisture accumulation are suitable conditions for bacterial and mold growth. Especially mold is associated with increased (>50%) humidity levels. Within only two days, mildew can propagate and release spores into the environment. The spores can cause allergic reactions and asthma, even in healthy persons. To avoid the growth and propagation of molds like *Aspergillus*, it is important to dry clothes after washing in an appropriate time frame. The dangers of mold produced mycotoxins are comprehensively reviewed elsewhere [66]. However, the spores themselves do not carry mycotoxins; the more dangerous part is the spore cell wall, that can cause immunoreactions upon exposure. This is especially the case for persons already suffering from asthma or allergies. The pollutants of microorganisms are not bound to surfaces alone (such as to the ceiling, or walls), they can also be distributed throughout the air by attaching to dust and other particles. That is why it is important to keep the indoor environment free from dust. Overgrowth of bacteria due to unclean conditions (such as standing water) can give rise to odor, and in the case of overgrowth of pathogens, may cause various diseases, including lung infection. Some important bacteria in the context of indoor pollution are *Staphylococcus aureus*, *Streptococcus pneumoniae*, and *Mycobacterium tuberculosis* [66]. Of course, a number of other non-pathogenic bacteria populate the human skin, and are an essential part of the gastrointestinal tract.

It is known that houseplants remove CO_2_, and in turn, release oxygen and water. There have been trials to use indoor plants to reduce some VOC compounds, such as toluene, benzene, and xylene [67]. Especially in the context of extended space travel, the use of plants is of high interest, and research in that area has already been initiated [68]. These studies came to inconclusive results, probably because the uptake of VOCs by plants is very slow, and only insignificant amounts were taken up [69,70]. It was shown that removal through passive and active ventilation in houses would remove pollutants faster and more efficiently than through plants. Despite these findings, some plants are recommended for removing biological and chemical pollutants, namely, the English ivy, Boston fern, and aloe vera. The plants probably remove the VOCs through absorption on their leaf surfaces. On the other side, there are recommendations not to keep plants that generate high moisture indoors, due to their support to increase humidity levels, which would result in mold growth and subsequently cause respiratory diseases [71].

Besides keeping the indoor environment clean, alternative methods to prevent microbial growth are provided by the heating, ventilation, and air-conditioning (HVAC) industry that offers indoor air recirculation systems, capable of trapping and removing microbes, pollen, and dust particles, and adjust the air moisture to the comfortable level of ~50%. Humidity control ensures a comfortable living environment indoors while making it difficult for molds to propagate. Additionally, the air pressure can be kept slightly positive, to keep the outdoor pollutants away and reduce their infiltration.

### 3.2. Outdoor Air Pollution

While the indoor air quality can be monitored, and improved by adjusting the buildings’ architecture, air filtering, and other methods, we have limited control on outdoor air quality. It is thus important to understand possible sources of contamination, available monitoring methods, and how to avoid inhaling harmful airborne substances.

The sources of gases and particles present in the air can be roughly classified into two major categories—natural (non-anthropogenic) and human-made (anthropogenic).

#### 3.2.1. Natural (Non-Anthropogenic) Pollution Sources

As mentioned earlier, many natural sources are polluting the air. Radon gas escapes from the Earth’s ground and can accumulate in confined areas. In some places on Earth, such as Siberia, methane (CH_4_) is released from the permafrost as a consequence of the temperature rise induced by global warming. Also, large herds of animals, such as cattle, may release considerable amounts of methane. Naturally occurring forest fires release carbon dioxide and carbon monoxide. CO_2_ is a naturally occurring gas in the environment, necessary for the growth of plants [72]. These wildlife fires also cause smoke and haze. Especially lightning-triggered fires are common in the forests of northern Canada in summer. Volcanic activity produces not only ash particles, but also sulfur, sulfur dioxide (SO_X_, SO_2_), chlorine, and other toxic gases in high quantities. In some regions, the vegetation can be responsible for seasonal haze. Plants release VOCs, which react with SO_2_, NO_X_, and organic carbon compounds, also contributing to the formation of haze [73]. Especially oak, willow, poplar, and black gum trees are known to produce large amounts of VOCs [74].

Interestingly, the salt spray over the oceans is the strongest contributor to the aerosols and particulates found in Earth’s atmosphere, and thus, the main source of non-anthropogenic generated aerosols. [75,76]. Sea spray consists of sodium chloride (NaCl), with some magnesium (Mg^2+^), sulfate (SO_4_^2−^), calcium (Ca^2+^), potassium (K^−^), and other components present in ocean water. Reports explain how the sea salt in the air removes the pollutants in the atmosphere via cloud forming processes [77]. On the other hand, there are reports on the possibility of secondary product formation from sea salts. The escaped chlorine from sea salt aerosol particles can promote HCl formation, leading to the formation of secondary pollutants, such as ozone or nitryl chloride (ClNO_2_) [78].

Dust storms (or in the context of the desert, sandstorms) are one of the naturally occurring pollution sources, with serious impact on air quality, and thus, human health, especially in Africa and the Middle East [76]. The blow of strong winds can detach and release mineral particles and other sand or soil particles from the Earth’s crust, forming aerosols over large areas of land [79]. The main terrestrial sources of airborne dust are the dry lands around North Africa and the Arabian Peninsula [80]. The dust storms are also formed in various Asian countries, including Iran, India, Pakistan, and China [81]. These fine dust particles are blown over long distances, even hundreds and thousands of kilometers. The high-speed surface winds disturb the ground in the deserts of Mongolia, northern China, and Kazakhstan, carrying dust particles over China, and even as far as Korea and Japan. The so-called yellow dust storm (also known as Asian dust storm) is prevalent during late winter and springtime in these regions [82]. It is possible to predict and follow sand storms. One of the best measures to prevent exposure to pollutants from dust storms is to stay indoors. Nevertheless, after sandstorms, many patients will visit doctors at the hospital for pharyngeal diseases, such as allergies and asthma [83]. Dust particles are known as potential carriers of viruses, bacteria, and fungi [84]. Together with soil particles, other toxic pollutants may be carried over by dust storms too. These pollutants include herbicides, pesticides, antibiotics, heavy metals (arsenic (As), lead (Pb), cadmium (Cd), mercury (Hg), chromium (Cr), zinc (Zn), copper (Cu)), and others [85]. 

#### 3.2.2. Human-Made (Anthropogenic) Pollution Sources

Biological systems were always exposed to non-anthropogenic airborne pollutants throughout evolution, and might thus have developed mechanisms to counteract possible detrimental effects. For example, forest fires and volcanic eruptions are non-anthropogenic sources for particulate matter (PM) in the biosphere, thus entering the respiratory tracts of animals since they evolved [86]. Airborne viruses have challenged the immune systems of living organisms throughout their evolution, a process that is ongoing [87]. With the discovery of fire usage, humans started to burn wood to cook, to keep themselves warm, to keep away predators and more. With the development of agriculture, the land was cleared by igniting forest fires, a practice that is ongoing in the Amazonas region or Indonesia. Therefore, air pollution can be regarded as something that accompanied humans from their earliest history until today. However, the amount and composition of airborne pollutants has changed due to anthropogenic activities, and might thus pose a challenge to human health [88].

In some developing countries, coal combustion is the primary method for heating homes and supplying energy. Coal mining and burning contributed to the famous hazes in England and the USA with the beginning of the industrial revolution, a problem that is still present in some developing countries. Power plants utilizing fossil fuels, such as petroleum (oil) fuel or biomass (dung, wood, and crop waste), also contribute to smoke emission. Industrial processes burning coal or petroleum regularly emit SO_X_ and SO_2_, as some coal and petroleum fractions contain sulfur compounds. The released SO_2_ can be further oxidized in the air (for example in the presence of NO_2_ as a catalyst), thereby forming sulfuric acid (H_2_SO_4_), resulting in acid rain.

Most vehicles, including cars, aircraft, and marine vessels, utilize non-renewable energy sources, such as the petroleum products gasoline or diesel [89]. The emissions from motor vehicles are one of the leading contributors to air pollution [90]. Their exhaust contains not only CO_2_ and water, but also many other toxic, cancerogenic gases and small particles, capable of penetrating deeply into lungs. Incomplete combustion of fuel or coal produces CO that can be found in the exhaust of vehicles, factories, but also furnaces meant for heating homes.

Methane (CH_4_) can be generated from waste deposits in landfills. If methane is not collected, it escapes into the atmosphere. VOCs can be emitted by sewage and garbage, but also by wastewater treatment plants and other industries. Aromatic VOCs, such as toluene and xylene, are known to be carcinogens. Other VOCs are sensed as odors that may severely impair the living comfort of humans. When the two primary pollutants nitric oxide (NO_x_) and VOCs mix and react in the air, secondary pollutants, so-called strong oxidants like ozone (O_3_) or even the stable oxidant peroxyacetyl nitrate (C_2_H_3_NO_5_), can be formed. As we discussed previously, strong oxidants can damage the mucosa and other human tissues.

Another class of pollutants with significant impact are persistent organic pollutants (POPs). POPs are organic compounds that have been and continue to be synthesized in huge amounts, as pharmaceuticals, industrial chemicals, solvents, and pesticides. POPs include compounds such as polychlorinated biphenyls (PCBs), that were used as heat exchange fluids, or dichlorodiphenyltrichloroethane (DDT), which was widely used as insecticide during World War II (WWII). POPs are resistant to environmental degradation through chemical, biological, and photolytic processes, and thus, tend to accumulate in the environment [91,92].

POPs are typically halogenated organic compounds, and are capable of entering the gas phase at environmental temperatures. Further, due to their volatility, POPs can easily enter the atmosphere from different sources, such as soils, vegetation, and water. These pollutants are resisting breakdown reactions in the air, and travel long distances before being re-deposited [93]. The long-range transportation of resistant POPs distributes these compounds as far from their origin as Antarctica and the Arctic Circle [94]. In higher organisms, POPs can accumulate in fatty tissues due to their high lipid solubility [95,96]. Some POPs are carcinogenic, some disrupt the endocrine system, and there are synergetic effects known for several POPs affecting the human body [97]. Exposure to POPs may cause chronic illnesses (obesity, diabetes), cancer, developmental defects, and even death [98]. Currently, there is a worldwide ban on the production and use of POPs [95].

Military related nuclear weapon tests, space and rocket science, research on toxic gases and biological warfare, further contribute to anthropogenic air pollution, including radioactive pollution.

#### 3.2.3. Aerosols and Particulate Matter

It is estimated that aerosols of anthropogenic origin currently account for approximately 10% of our atmosphere. Non-anthropogenic formed aerosol particles tend to be smaller than particles originating from human activity [79,99]. Smaller (<1 µm) and lighter particles tend to stay longer in the air, even for weeks, until they are removed from the atmosphere by precipitation. Larger particles (>10 µm) will settle to the ground by gravity within hours. Most of the particles that enter the respiratory tract of humans are filtered via cilia and mucus, and removed on the mucociliary escalator [100]. If swallowed, a rather small amount of these particles may be subsequently absorbed through the gastrointestinal tract, while most of them will be excreted [101]. In general, epithelial barriers in the body (skin, lungs, gut, and so on) are very restrictive to nanoparticle entry, as long as they are not compromised by injury or inflammation. Some particles penetrate beyond the larynx, and only very small particles reach and enter the bronchi and lungs. Usually, particles smaller than 10 µm will reach the bronchioles or alveoli [102].

While the term aerosol encompasses the mixture of solid and liquid matter, PM refers exclusively to solid particles that might be found in the atmosphere [103,104]. The atmospheric dust dissolved in the atmosphere can carry particles as small as 10 nm, which is in the size range of small viruses. Some examples of where PM can be found in significant amounts are tobacco smoke, soot, or smog. PM can impact the global climate and adversely affect human health. The World Health Organization (WHO) designated airborne PM as a Group 1 carcinogen [105]. Studies showed that there is no safe level of the exposure with PM and with any concentration increase of PM in the air, the cancer rate increases proportionally [106,107].

The smallest particles with diameters less than 100 nm (nanoparticles or ultrafine particles (UFP)) can further penetrate the alveolar–capillary membrane and migrate into diverse organs, including the brain [108,109,110,111]. Such small particles, also termed diesel particulate matter (DPM), are prevalent in the exhaust from diesel engines, even in the latest and more advanced ones [112,113]. Emissions from diesel vehicles are thus considered significantly more harmful than those from petrol vehicles [114]. Therefore, it seems recommendable for people that suffer from allergy or asthma to drive a car that consumes petrol rather than diesel (or even better, an electric car). Small sized particles possess a high surface-to-volume ratio, i.e., a given quantity of nano-sized matter has a larger surface area than the same quantity in bulk form. Many carcinogens, like benzopyrenes, can adsorb on the small particles’ surface, and are carried through air, eventually reaching the human body. A higher prevalence in the development of cancer was noticed early in towns with highways for diesel-powered trucks when compared to more rural areas [115]. Truck drivers also have higher cancer rates, due to the diesel pollution, when compared to other job categories not directly related to the diesel engine exhaust [116]. Interestingly, the long-haul truck drivers were at lower cancer risk compared to the short-haul truck drivers. The long-haul drivers shut down the windows, which gives an additional protection, as the incoming air is filtered through the air filter. The short-haul truck drivers, in contrast, leave their windows open and are thus exposed to the toxic exhaust originating from their vehicles [117].

The exposure to PM can have a detrimental health effect, and is suspected of harming pregnant women and unborn children. PM can cause oxidative stress, leading to inflammation. Other effects on pregnancy include endocrine disruption and impaired oxygen transport to the placenta, leading to a lower birth weight [118,119], congenital disabilities (birth defects), premature delivery, or even premature death [120,121]. However, the studies related to a lower birth weight are inconclusive, and more research is needed [122].

In vivo studies on animals and adult humans have demonstrated that the exposure to PM leads to the development of asthma, respiratory diseases, lung cancer [107], and cardiovascular disease [123]. In some countries, efforts to control the diesel exhaust levels go as far as to ban diesel vehicles totally from city centers (examples include Paris, Mexico City, Madrid, and Athens) [124].

The recognition of the dangers posed by particulate matter has led to monitoring their levels in the air. Other prevention measures include the installation of necessary filters in industry, and efforts to minimize the number of conventional cars on the streets. In India, public buses now use compressed natural gas, which helps to eliminate city smog [125,126]. 

Many countries have their air quality index (AQI) definition, and monitor the air pollutant concentration over a specific period (such as every hour). Different countries have different approaches to calculate the AQI. Most often, the AQI score is computed, or simply assigned to the highest of individual PM2.5 or PM10 pollutant scores (I-AQI). For example, in China, air monitoring stations are located in every bigger city or district. The data from these stations are made available online, and include values such as PM10, PM2.5, SO_2_, NO_2_, CO, and O_3_ (Figure 1). The exact nature of pollutants that are monitored differs between countries. For example, in India, besides the six atmospheric pollutants monitored, the concentrations of ammonia (NH_3_) and lead (Pb) are also monitored. Different countries set different limits for particulates in the air (PM10 and PM2.5). Ideally, there should be no PM in the air at all. It can be expected that a decline in the use of fossil fuels, accompanied by a transitioning to renewable energy sources, will have remarkable effects on the global air quality; a trend every nation, every human being, and all animals will benefit from. 

## 4. Emerging On-Chip Technologies for the Air Quality Measurements

On-chip technologies enable the creation of fast, precise, simple, portable, low-cost gas sensing platforms with wide application areas, including air quality monitoring [127,128]. The most widely known electrochemical sensing principles, based on charge generation or conductivity changes, are already available on the market and implemented as on-chip products [129]. At the same time, gas sensors based on MEMS technology are superior to other sensor types regarding selectivity, decreased power dissipation, lower operating temperature, and quicker response. Moreover, some of the “pure” MEMS sensor elements, such as bridges, cantilevers, membranes, etc., can be fabricated by the well-established complementary metal-oxide-semiconductor (CMOS) technology [2]. This provides unmatched opportunities regarding technology advances and price lowering for the integration of the sensing chips with microelectronics, and even allows for the co-fabrication of electronic circuitry and sensor architecture.

Using MEMS structures for air quality sensing means that gravimetry needs to be employed in one way or another [130]. Gravimetric on-chip detection can primarily be used to sense comparatively larger particles in the gaseous environment: dust, microparticles, nanoparticles, and aerosols and liquid microdroplets trapped within the mechanical structure. Also, recent developments demonstrate the suitability of gravimetric principles for highly selective sensing of low molecular weight gases, such as CO_2_ [131,132]. MEMS gas sensors deliver the promise of higher sensitivity, as they are more selective in distinguishing between various gases. Their advantages are demonstrated by reports of a reached sensitivity of 4 Hz/ppm for CO_2_ by using capacitive ultrasound transducers (CMUT) type sensor [132]. More convenient metal oxide electrochemical sensors have a detection limit in the range of hundreds of ppm for CO_2_, and for this, require elevated temperatures [133,134].

Gravimetric sensing of gas molecules requires the development of a functional layer, which selectively binds or adsorbs gas molecules. A primary example of a gravimetric detection principle is quartz crystal microbalance, which provides mass detection by analyzing changes in the resonance frequency of a crystal induced by the presence of certain molecules on the surface of the crystal [135,136]. The common readout solution for this sensor type is to connect the crystal as an electromechanical resonator to an oscillator circuit. This provides the possibility for efficient, low noise, and compact detection [130]. Besides, of classical implementation of the gas sensing MEMS, which are based on piezoelectric materials, there is no requirement for piezoelectric properties of materials for the resonance MEMS, based on cantilevers [137,138] or membranes [131,132,139] that allow for a capacitive readout. Moreover, some of the CMUTs feature vacuum-backed membrane structures and therefore are advantageous because of potentially higher working frequencies and higher resonance quality compared to other MEMS structures, which do not have vacuum-backing. Better resonance quality provides sharper frequency pointerand makes lower frequency shifts detectable, while higher oscillation frequency is important, because the absolute frequency shift is greater at higher resonance frequency for the same added mass [131], so the sensitivity potential is greater.

Nevertheless, the need for materials that specifically bind the molecules of specific gases to the surface slows down the development of gravimetric sensors. For example, there are only a few candidates to establish the functional layers for CO_2_ sensing [131,132], and only limited progress regarding MEMS-based gravimetric sensing of other hazardous gases, such as NO_X_, SO_2_, or VOCs. Electrochemical sensors utilizing conductive polymers [140,141], metal oxides, their combinations, and nanowires [142,143] represent proven technologies for the sensing of these gases. Commercially available electrochemical on-chip sensor technology is based on a catalytic action, often driven by auxiliary temperature. The micro-hotplate and micro heaters solutions, partially using advantages of MEMS fabrication technology, were proposed to lower the energy demands for these catalytic sensors [144]. This class of gas sensors still suffers from stability and degradation issues [145], and still needs improvements regarding their energy efficiency [146].

## 5. OoC for Assessing Health Effects

### 5.1. OoC Technology for Modeling Healthy and Pathological Lung Function

Inhalation studies using animals are the current gold standard when potential risks of airborne pollutants need to be assessed. However, the translation of results gained in animal studies to humans is hampered by differences in lung physiology and ventilation characteristics. These parameters can influence the rate of particle deposition, and thus, the cellular (effective) dose [147]. For better comparability and the identification of safety thresholds, humanized systems that allow for control of the applied dose and exact determination of the cellular dose would be needed. Here, OoC technology might be added as a valuable tool in the animal-free testing of short and long-term effects on the respiratory system by exposure to airborne pollutants.

The lung is a tissue with a highly complex physiology. The trachea, bronchi, and bronchioles form the conductive airways, while the alveoli can be regarded as the smallest functional unit facilitating the actual gas exchange. The paranasal sinuses are also considered to be part of the human airways, yet they are not involved in the process of breathing and gas exchange. The distinct compartments of the lung have different structural characteristics, such as stiffness and cellular composition, that determine their respective function [148]. Furthermore, the lung has features that are quite distinctive from other tissues. These include a high elasticity that facilitates air exchange through breathing, an air–liquid interface, and high gas permeability for gas exchange in the alveoli. The lung is also a barrier tissue that is meant to prevent foreign bodies and pathogens from entering the body while ensuring the exchange of O_2_ and CO_2_. Rather large particles and pathogens are removed through mucociliary transport, while pollutants such as very small particles and pathogens that reach smaller compartments, like the alveoli, are removed by macrophages [149]. The alveoli are the lung compartment that is of key importance for gas exchange, but also, the transfer of airborne pollutants into the blood stream. Hence, most published OoC do not try to recapitulate the complexity of the whole lung, but rather, the alveoli as the interface between air and blood stream. 

**Figure 2:**
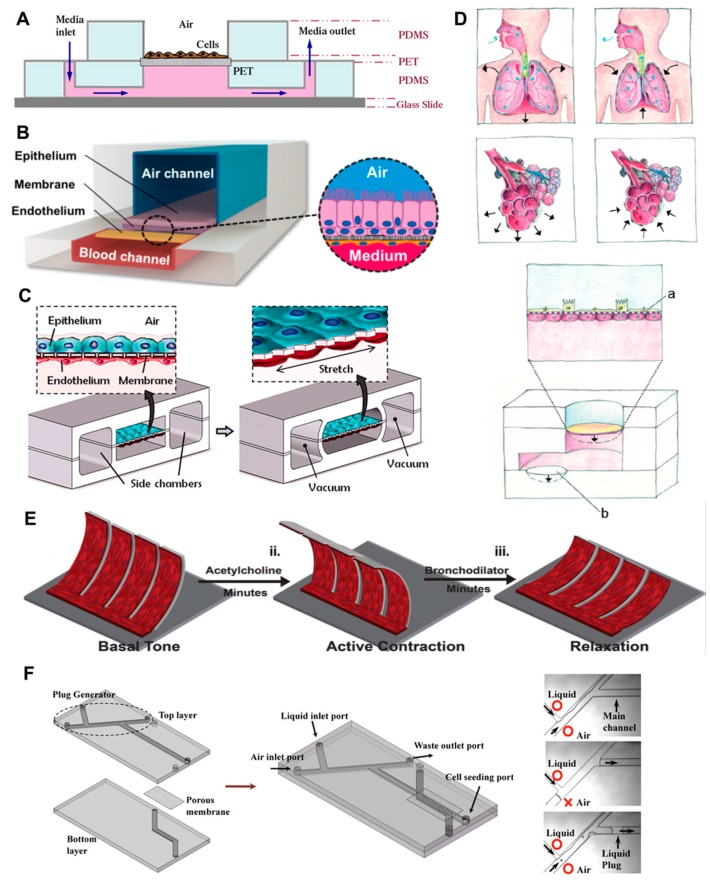
Different lung-on-a-chip design principles. (**A**) The cross-sectional view though the microfluidic device shows an early attempt to recreate lung physiology by introducing an air–liquid interface to the cells. Reprinted from [150], with permission from Elsevier; (**B**) 3D cross-sectional view illustrates the use of an epithelial and endothelial cell line separated by a porous membrane that divides air and a media channel. Reprinted by permission from Macmillan Publishers Ltd: Nature Methods [151], copyright (2015); (**C**) Building on similar design considerations, the stretchability of the separating membrane is introduced by using vacuum chambers at both sides of the actual compartments for cell growth. From [154], reprinted with permission from AAA; (**D**) Since breathing introduces 3D multidirectional mechanical stress to the alveoli in human lung, a system was introduced that simulates this stretching more close than unidirectional stretching does. [156]—Published by The Royal Society of Chemistry under creative commons Attribution 3.0 Unported License; (**E**) Schematic depicting engineered bronchial smooth muscular thin films adopting (i) basal tone, (ii) preconstriction, and (iii) bronchodilator-induced relaxation. Reproduced from [158] with permission from The Royal Society of Chemistry; (**F**) Shown is a schematic of the microfluidic airway model and photomicrographs of the liquid plug generation. Reprinted from [160], © Springer Science+Business Media LLC 2011, with permission from Springer. PET: Polyethylene

First attempts to recreate the alveolar interface focused on engineering principles, rather than an exact recapitulation of biology, while later systems show a higher sophistication on the cellular level. For example, Nalayanada and colleagues introduced an early approach to recreate the alveolar air–liquid interface. Here, A549 human alveolar basal epithelial cells were cultured on top of a membrane with 0.4 µm pore size, while the lower side of the membrane had access to a fluid channel filled with media (Figure 2A). The cells themselves had direct contact with ambient air, which resulted in the formation of a surfactant layer, similar to the physiologic situation in the alveoli [150]. Building on similar design considerations, others have introduced designs that encompass the use of epithelial and endothelial cells separated by a porous membrane (Figure 2B). The membrane itself divides two channels, with the upper one experiencing air flow, and the lower one, media flow [151,152,153]. This general design can be further developed to enable cyclic stretching of the separating membrane, therefore simulating alveolar stretching in response to breathing movements (Figure 2C) [154]. While being an elegant example of an organ-on-a-chip system, the lung-on-a-chip of Huh and colleagues is still an approximation of the physiological counterpart, due to different dimensions, the use of cell lines, and an artificial membrane for keeping apart the two cell layers. Also, the unilateral stretching of the membrane only partially represents the 3D mechanical strain found in spherically shaped alveoli [155]. To recreate physiological strain patterns, Stucki and colleagues introduced a microfluidic system that recreates the widening of alveoli in lung induced by stretching of the diaphragm [156]. In their model, primary cells are seeded on a thin Polydimethylsiloxane (PDMS) membrane that is stretched in all three dimensions by a micro-diaphragm that is electro-pneumatically actuated, therefore recreating the physiological strains that cells experience in the alveoli more closely (Figure 2D). Douville and colleagues presented a similar approach, with the addition that the artificial alveoli were partially flooded with media, to additionally simulate the surface tension stress experienced by cells through the cyclic propagation of the air–liquid interface [157].

Systems that recapitulate other anatomical structures of the lung were also published. For example, Nesmith and colleagues developed a human airway musculature on a chip that allows modeling the constriction and dilation of bronchi [158]. This system mimics human bronchial smooth muscle (BSM) lamellae, the simplest structural and functional unit of the airways. For this, bronchial smooth muscular thin films were engineered by seeding primary BSM cells on elastic PDMS cantilevers. Once the BSM cells contract, the thin film bends, leading to a reduction of the tissues’ curvature radius, thus modeling bronchoconstriction (Figure 2E). Other approaches focus on modeling physiological parameters, rather than anatomical structures found in the lung, such as differences in oxygenation. In their microfluidic model, Skolimowski and colleagues recapitulate the anaerobic, micro-aerobic, and highly aerobic environment found in sinuses, trachea/bronchi, and alveoli, respectively [159]. These differences in oxygenation play an important role during infections of the airways, since oxygen is needed for proper immune cell function, and also, limits bacterial growth according to the pathogens respiration mode (aerobic, facultative anaerobic or anaerobic). Therefore, antibiotic treatment of infections in different airway compartments can be modeled with this system. In various diseases of the lung, a deficiency of pulmonary surfactant can give rise to a disturbed liquid lining of the small airways, and to the formation of occluding liquid plugs. The propagation of liquid plugs devoid of surfactant can lead to large mechanical forces experienced by cells of the airways. In their model, Tavana and colleagues present a microfluidic model that recapitulates airway architecture, simulates physiologic levels of pulmonary pressures, and allows for the cultivation of airway cells (Figure 2F). The latter enables the study of A549 cells exposed to repeated liquid plug propagation, and therefore, to mechanical shear forces that can rise to harmful levels [160].

In the studies mentioned above, the use of endothelial and epithelial cell lines, and the feasibility of using primary human cells for lung-on-a-chip models, were both demonstrated. Cell lines and primary cells have their respective advantages and drawbacks, yet in the case of lung, it should be noted that biopsies from healthy donors are difficult to acquire, expand, and maintain [148]. This relative scarcity of suitable donor material is a limiting factor in establishing the widespread use of lung models based on primary cells.

### 5.2. OoC Technology for Modeling Lung Pathologies and Pollutant Exposure

Notably, a number of diseases linked to the exposure to pollutants were successfully modeled in microfluidic systems by using the discussed lung-chips. Various diseases, including asthma, are accompanied by lung inflammation that includes the infiltration of polymorphonuclear eosinophils. In chronic asthma, these cells contribute to pathological airway remodeling by releasing toxic proteins that cause changes in pulmonary surfactant and damage to airway tissue. In their study, Punde and colleagues were able to mimic the migration of eosinophils across the blood–tissue barrier in the airways, and also, to study the effect of a secreted factor known as eosinophil cationic protein [152]. Although these mechanisms only represent one aspect of the asthma etiology, the proposed experimental set up allows for their study in unprecedented detail. 

Inflammatory processes also accompany the progress of COPD. In their work, Benam and colleagues used cells from healthy and COPD donors in their lung-on-a-chip device [151]. They were thus able to recreate inflammatory processes and neutrophil recruitment, both being characteristic in the development of COPD. Most notably, it was possible to use this system for the screening of new anti-inflammatory drugs that might contribute to the treatment options regarding COPD. Building on this model, the effect of cigarette smoke on cells from healthy and COPD donors was investigated [18]. For this, a device was engineered that mimics respiration characteristics of a human smoking a cigarette. The smoke was then transferred into the airway compartment of the lung-on-a-chip to successfully recreate smoking associated pathologies. This approach could, in theory, be applied to other sources containing airborne mixed pollutants, such as exhaust fumes from diesel engines.

Using their breathing chip design, Huh and colleagues were able to recreate interleukin-2 (IL-2) induced vascular leakage that eventually leads to pulmonary edema. In addition, it was demonstrated that mechanical cyclic stretching, due to breathing, plays an important role in the development of IL-2 induced lung edema [161]. Since IL-2 is administered in cancer patients, severe side effects should be avoided. Here, the authors were able to identify new treatment options for the pulmonary toxicity of IL-2, thus demonstrating the feasibility of using OoC technology for gaining better mechanistical understanding of pathologies and subsequent drug screening. The same breathing chip design was used to demonstrate that nanoparticle translocation from alveoli to the blood stream requires cyclic stretching of the epithelial–endothelial barrier [154]. This study shows the value of including tissue specific physical parameters into organ-on-chip devices, and also highlights how in vitro models can be expanded to closer recreate the in vivo situation.

Other disease models include hereditary lung diseases, such as cystic fibrosis, or simulate the occlusion and reopening of airways which accompanies the deficiency or dysfunction of pulmonary surfactant during various lung pathologies [159,160].

In summary, the use of OoC technology in the context of investigating lung function and pathology is feasible. Factors that might limit the full reconstruction of organ function in lung models include the physiological complexity of the tissue of interest, and the low availability of primary cell material. Since lung is a barrier tissue, the addition of immune cells, such as macrophages, would help to increase the physiological relevance of the respective models. However, current models are already useful for investigating the effect of airborne pollutants on alveolar cells and their potential transfer into the blood stream, and thus, the possibility of their system-wide distribution.

### 5.3. PM2.5 Particles Penetrate Deep into Tissues

**Figure 3:**
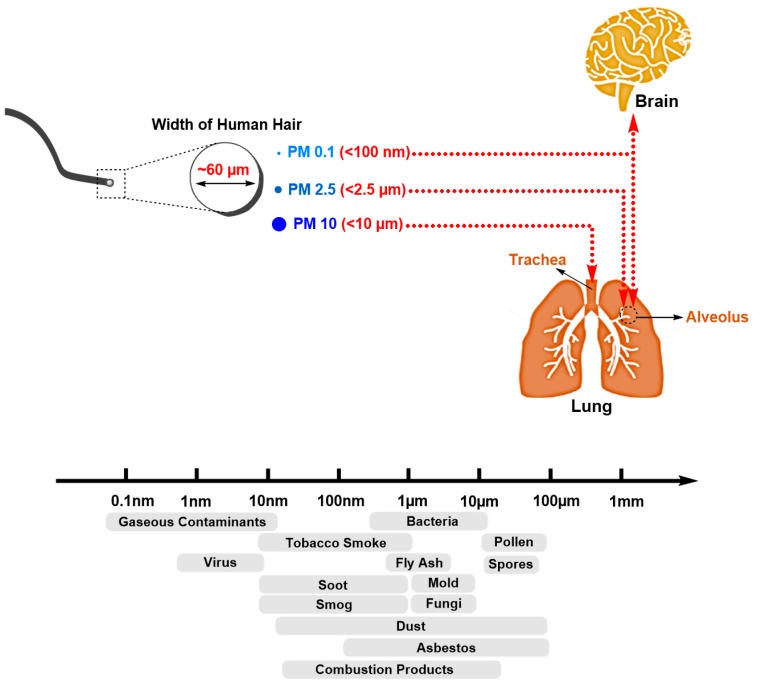
One can visualize how small the air particles are by comparing them to the human hair. The hair cross-section diameter being ~60 µm, about six PM10 particles and 24 PM2.5 particles could extend over the hairs cross-section perimeter. PM2.5 (or fine PM), tend to penetrate into the gas-exchange regions of the lung (alveolus).

PM2.5 particles are especially harmful because they can easily enter the alveoli and cross the membrane of lung cells, eventually accumulating in the respiratory system. It is estimated that 75% of <2.5 µm diameter particles, and ≈100% of <2 µm particles, will reach the alveoli (Figure 3).

When PM2.5 enter the lung, the particles will have a variety of possible ways to migrate further [162,163,164]. One way is a migration into the bronchial lung parts, where the particles will interfere with gas exchange, causing asthma, bronchitis, cardiovascular disease, etc. When pulmonary macrophages phagocytose the particles, they might enter the lymphatic system. Additionally, the PM2.5 can also break through the lungs’ blood barrier, and enter the blood circulation system, thus reaching other organs. Also, PM2.5 particles may be retained in the lungs for long periods of time. The particles can interact with alveolar macrophages and pulmonary epithelial cells, stimulate the release of a variety of cytokines, and lead to inflammation and fibrosis.

### 5.4. Cancer Types Caused by PM

PM2.5 particles are considered as potential carriers of diverse harmful substances causing a variety of diseases [165,166,167]. The effect of PM2.5 particles on human health depends on the physical and chemical properties of the PM, especially on the chemical composition. The chemical composition of PM2.5 particles can be complex. PM2.5 may contain heavy metals, such as lead (Pb), cadmium (Cd), arsenic (As), mercury (Hg), and chrome (Cr); but also, organic compounds, such as polycyclic aromatic hydrocarbons (PAH). PM2.5 particles can cause damage to the respiratory and the cardiovascular systems, leading to coughing, dyspnea, asthma, chronic bronchitis, arrhythmia, etc. [164,167,168]. Also, some reports showed that PM2.5 might cause severe cancer, such as lung cancer, uterine leiomyomata, and leukemia [105,169,170,171].

**Figure 4:**
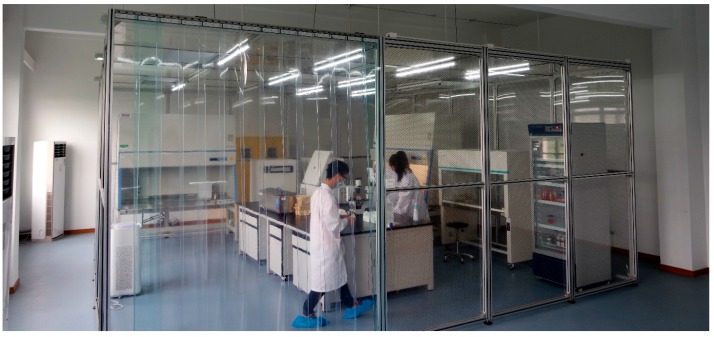
The Shanghai University (SHU) iSmart Institute cell culture room for the organ-on-chip (OoC) experiments. The area is isolated, with the high efficiency particulate air (HEPA) filtration, temperature and humidity control. The air pressure inside is kept positive to keep away the outside air. The cell culture room has an area of 30 m^2^ (5 × 6 m) and accommodates the instruments necessary for work: refrigerators, freezers, cell culture incubators, biological safety cabinet, microscope, and chip setup with fluid control.

The development of cancer, such as lung cancer, is known to be a long process. The currently high prevalence of lung cancer may be the result of the long-term exposure to the polluted environment (such as exposure to traffic exhaust fumes and combustion products) and risk factors arising from an unhealthy lifestyle (such as obesity and smoking). Among other industrial countries, China is known for a high incidence of lung cancer. The reason for this has always been considered to be found in long-term excessive smoking in the past. However, in the recent years, with significantly increased PM2.5 values in many Chinese cities, the carcinogenic nature of PM2.5 attracted a wider attention. Although the increase in the incidence of lung cancer cannot be attributed entirely to the increased PM2.5 values, some studies have shown that polycyclic aromatic hydrocarbons (PAH) and heavy metal ions in PM2.5 play an important role in the occurrence of lung cancer [172]. More experimental and case studies are required to confirm this hypothesis (Figure 4).

### 5.5. Pharmaceutical Drugs to Treat Lung Cancer

For the treatment of cancer, it is important to understand the histological classification of lung cancer [173]. Previously, lung cancer was classified either as small cell lung carcinoma (SCLC) or as non-small cell lung carcinoma (NSCLC). The current classification of lung cancer is more refined, but the SCLC and NSCLC classification is still used in the clinical setting. At present, the SCLC and NSCLC treatment programs utilize a combination of two or more chemotherapy drugs. One of the common drugs is platinum-based, such as cisplatin, carboplatin, and others [174]. The SCLC treatment often uses CRT-11 (irinotecan) and cisplatin, and for NSCLC treatment, cisplatin/carboplatin is combined with either paclitaxel or gemcitabine [175]. Also, as a targeted drug, gefitinib (which is an epidermal growth factor receptor tyrosine kinase inhibitor), is used for the treatment of advanced NSCLC [176].

## 6. Summary

Our body is in direct interaction with the air that surrounds us from birth until death. Throughout our entire evolution, humans have learned to cope with natural pollution sources, such as salt spray from the oceans, dust storms, wildfires, volcano eruptions, etc. Usually, natural pollution sources are temporal and do not last very long (such as wildfire), or are seasonally reoccurring (such as dust storms). Although natural pollution produces rather small particles [79,99], which can be more deeply inhaled into lungs, and are therefore more dangerous, the natural pollutants are overall less toxic, regarding their chemical formula, compared to those of anthropogenic origin.

Anthropogenic pollution affects the air quality both indoors and outdoors. Here, burning fossil fuels can be regarded as the main source of pollutants. Aside from SO_2_, NO_X_, and other exhaust gases, there are many anthropogenic pollution formulations, such as POPs, that are persistent, and thus will affect our environment in the long-term.

Of course, it is of high importance to identify sources of pollutants and to follow their migration patterns. On the global scale, large area sand storms, industrial gases, and other pollutants can be monitored with the help of satellites. Satellites can be equipped with various sensors, capable of detecting and quantifying the size and chemistry of particles. For example, a moderate resolution imaging spectroradiometer (MODIS) is used to gather information on various gases, including formaldehyde (CH_2_O) [8,10]. On the ground level, the air quality in many cities is monitored with the help of meteorological stations. The data can be accessed in real time online; mobile phone applications are also available. For example, The World Air Quality Index project provides information from more than 70 countries by collecting and summarizing air pollution data from 600 major cities with more than 9000 stations in total (Figure 1) [11].

Indoor air can be monitored for various pollutants, such as CO_2_ and VOCs, while conditioning systems can be adjusted to improve air quality. Additionally, the air inside houses or cars can be filtered through various filters (such as a High efficiency particulate air (HEPA) filter). There are many tools to monitor and improve air quality. The best solution is to understand the source of a particular pollutant and minimize its output. There are many examples of new and untested materials being introduced to our environment prematurely, and only in later steps, their hazardous potential is recognized, eventually leading to their complete ban. That happened with asbestos, which was popular until 1975 [61], POPs [91,92], and others. Now, there are worldwide regulations in place, including a total ban on these substances. We predict the same may happen to the burning of coal and diesel fuel, as these fuels produce cancerogenic particles in the nanoscale size. We observe the constant progress towards a cleaner environment, fueled by the demands of an ever-increasing number of educated and concerned citizens who exert pressure on policy-making authorities. For example, in China, there are currently very few personal vehicles using diesel, with increasing popularity of silent and clean electric-powered cars and scooters.

Small detection chips are gaining popularity in sensing various gases, particle size, and chemical composition of the air. These chips are small, cheap, and can be included in mobile stations to provide data in real time [129,133]. Advances in MEMS and CMOS technologies allow for the utilization of gravimetric on-chip detection for CO_2_ and, with limitations in detection sensitivity, for NO_x_, SO_2_, or VOC gases [140,141]. New materials, such as a combination of metal oxides, nanowires, or conductive polymers, allow for the development of novel electrochemical sensors [143]. There are still some issues with stability and energy requirements, which we expect to be solved by using microheaters in combination with other technologies [145,146].

Aside from their overly negative effects on the environment, airborne pollutants pose a major hazard to human health. Our body is in constant contact with air. The main routes of exposure resulting in health risks are through the dermal (skin) or respiratory systems. The particle chemistry, size, and shape, have a major impact on the hazard a particular pollutant poses. The particulate matter PM2.5 and nanoparticles are especially dangerous, as these particles are capable of entering our blood stream, from where they may reach other organs [111]. Burning of incense in religious rituals, but also, burning agarwood (oud) produces smoke that people find pleasant and relaxing, despite the small particles present in the smoke. This phenomenon needs more investigation, as the continuous breathing of these special scents may even stimulate the immune system [177].

Gases like CO, CO_2_, and VOCs may affect the brain, causing headache and fatigue. PM2.5, O_3_, SO_2_, and NO_x_ affect the lung, causing respiratory pathologies. SO_2_ and NO_X_ additionally can cause cardiovascular illness. VOCs lead to skin irritation, cause nausea, and increase the risk of developing cancer. Various bacteria, parasites, and chemicals carried by dust particles may promote asthma and cause allergic reactions.

In particular, the pollutant effects are evident only after long-term exposure, and are difficult to recreate in conventional cell culture [108,178]. Here, OoC technology might help to bridge the gap between in vivo observations and in vitro experiments. For example, cancer formation utilizing human cells can be simulated on the OoC within weeks, while cancer formation after the exposition of the body to pollutants usually takes months to years.

It is obvious that air pollution will remain an important issue in the future. Microchip technology will gain more impact in the field of air monitoring, so that the pollution sources can be detected early, and exposure of humans can be minimized. We expect that substances proved to be a hazard to human health (such as asbestos, POPs, etc.) will be soon be completely banned worldwide. There are many new substances, including anthropogenic nanoparticles and novel polymers being developed and supplied to our market. Novel materials are always potential pollutants, and should be tested for their effect on organs and tissues by progressive utilization of OoC technology, instead of solely relying on animal testing and conventional cell culture experiments. This would enable better estimates on the toxicity of novel materials on human cells. Additionally, the chip technology can be used for the detection of various illnesses and cancer types. For example, more research is needed to detect and treat cancer early. OoC are very handy in here—human cells can be grown under various conditions and supplemented with different available pharmaceutical drugs, novel ones or their combination.

While humans are responsible for the anthropogenic activities and have the biggest impact on our shared biosphere, we would like to point out that all life forms are affected by the biosphere pollution. It is, therefore, our responsibility to minimize environmental pollution.

## Figures and Tables

**Table 1:** A non-exhaustive list of companies engaged in Organ-on-Chip (OoC) development or providing services in this field.

Company	Country	Developing the OoC Models	Providing Services or Chips in the Field of OoC
AxoSim Technologies	US	x	
CNBio Innovations	UK	x	
Emulate	US	x	
Mimetas	The Netherlands	x	
TissUse	Germany	x	
Hesperosinc	US		x
Hurel Corporation	US		x
InSphero	US		x
Microfluidic Chipshop	Germany		x
Micronit	The Netherlands		x
Molecular Devices	US		x
Neofluidics	US		x
Nortis	US		x
TARA Biosystems	US		x
